# Comparative cytogenetics of Serrasalmidae (Teleostei: Characiformes): The relationship between chromosomal evolution and molecular phylogenies

**DOI:** 10.1371/journal.pone.0258003

**Published:** 2021-10-07

**Authors:** Ramon Marin Favarato, Leila Braga Ribeiro, Alber Campos, Jorge Ivan Rebelo Porto, Celeste Mutuko Nakayama, Rafaela Priscila Ota, Eliana Feldberg

**Affiliations:** 1 Programa de Pós-Graduação em Genética, Conservação e Biologia Evolutiva, Instituto Nacional de Pesquisas da Amazônia, Petrópolis, Manaus, Amazonas, Brazil; 2 Centro de Ciências da Saúde, Universidade Federal de Roraima, Avenida Capitão Ene Garcêz, Boa Vista, RR, Brazil; 3 Coordenação de Pesquisas em Biodiversidade, Instituto Nacional de Pesquisas da Amazônia, Petrópolis, Manaus, Amazonas, Brazil; 4 Departamento de Biologia Estrutural e Funcional, Instituto de Biociências, Universidade Estadual Paulista “Júlio de Mesquita Filho”, Botucatu, São Paulo, Brazil; Natural History Museum of London, UNITED KINGDOM

## Abstract

Serrasalmidae has high morphological and chromosomal diversity. Based on molecular hypotheses, the family is currently divided into two subfamilies, Colossomatinae and Serrasalminae, with Serrasalminae composed of two tribes: Myleini (comprising most of pacus species) and Serrasalmini (represented by *Metynnis*, *Catoprion*, and remaining piranha’s genera). This study aimed to analyze species of the tribes Myleini (*Myloplus asterias*, *M*. *lobatus*, *M*. *rubripinnis*, *M*. *schomburgki*, and *Tometes camunani*) and Serrasalmini (*Metynnis cuiaba*, *M*. *hypsauchen*, and *M*. *longipinnis*) using classical and molecular cytogenetic techniques in order to understand the chromosomal evolution of the family. The four species of the genus *Myloplus* and *T*. *camunani* presented 2n = 58 chromosomes, while the species of *Metynnis* presented 2n = 62 chromosomes. The distribution of heterochromatin occurred predominantly in pericentromeric regions in all species. *Tometes camunani* and *Myloplus* spp. presented only one site with 5S rDNA. Multiple markers of 18S rDNA were observed in *T*. *camunani*, *M*. *asterias*, *M*. *lobatus*, *M*. *rubripinnis*, and *M*. *schomburgkii*. For *Metynnis*, however, synteny of the 18S and 5S rDNA was observed in the three species, in addition to an additional 5S marker in *M*. *longipinnis*. These data, when superimposed on the phylogeny of the family, suggest a tendency to increase the diploid chromosome number from 54 to 62 chromosomes, which occurred in a nonlinear manner and is the result of several chromosomal rearrangements. In addition, the different karyotype formulas and locations of ribosomal sequences can be used as cytotaxonomic markers and assist in the identification of species.

## Introduction

Serrasalmidae is a family of fishes endemic to the Neotropical region, which are distributed mainly in the Amazon, Orinoco and Paraná-Paraguay basins [1, 2], with lower representativeness in the São Francisco River basin and introductions reported in coastal basins [3]. Despite the occurrence of some species in environments such as rapids, these fishes typically inhabit lakes and floodplains [4]. It is the fourth most diverse family within Characiformes, with 101 valid species, distributed in 16 extant genera [5–7], with more than 70 species occurring in the Amazon basin [8]. These fishes have different feeding habits, and can be frugivorous, herbivorous, piscivorous, and lepidophagous (consumer of scales) [9–11].

Family monophyly is supported by numerous morphological synapomorphies, such as the presence of a ventral keel, composed of spines derived from modified abdominal scales, an anteriorly oriented predorsal spine, only absent in *Colossoma* Eigenmann & Kennedy 1903, *Mylossoma* Eigenmann & Kennedy 1903, and *Piaractus* Eigenmann 1903, and by the presence of interlocking teeth in premaxilla and dentary [12, 13]. Serrasalmidae has also been recovered as monophyletic in molecular phylogenies proposals [14–17].

The evolutionary relationships within the family have been the subject of several studies [15, 18–20]. Commonly the species have been recovered and grouped into three large clades: (i) the first, composed by genera lacking the predorsal spine; (ii) the second, comprising *Acnodon* Eigenmann 1903, *Mylesinus* Valenciennes 1850, *Myleus* Müller & Troschel 1844, *Myloplus* Gill 1896, *Ossubtus* Jégu 1992, *Tometes* Valenciennes 1850, and *Utiaritichthys* Miranda Ribeiro 1937; and (iii) the third, represented by *Catoprion* Müller & Troschel 1844, *Metynnis* Cope 1878, *Pristobrycon* Eigenmann 1915, *Pygocentrus* Müller & Troschel 1844, *Pygopristis* Müller & Troschel 1844, and *Serrasalmus* Lacepède 1803 [15, 19]. Kolmann et al. [20] named these clades as subfamilies Colossomatinae, Myleinae, and Serrasalminae, respectively.

However, the hypothesis of the intrafamilial relationships within Serrasalmidae using of Ultraconserved Elements (UCEs), included all genera and the greatest number of species, and proposed a slightly different classification [6]. The family is presently composed by two major clades: (i) Colossomatinae, represented by species lacking a predorsal spine, and (ii) Serrasalminae, with species having a predorsal spine, and divided into Myleini (pacus) and Serrasalmini (*Metynnis* plus piranhas). Despite these advances, the monophyly of some genera was rejected, as *Myleus*, *Myloplus*, *Pristobrycon*, and *Tometes* [6, 20].

Similarly, studies using the gene cytochrome c oxidase subunit I (COI) revealed that the diversity within Serrasalmidae is still underestimated. In the Brazilian Amazon, various lineages may represent new species, especially concerning *Myloplus* and *Serrasalmus* [21]. Two new species were recently described using the same marker, *Catoprion absconditus* Mateussi, Melo & Oliveira 2020 and *Myloplus nigrolineatus* Ota, Machado, Andrade, Collins, Farias & Hrbek 2020 [6, 7]. In the basins of the Paraná-Paraguay and Tocantins rivers, a species complex was detected in *Serrasalmus maculatus* Kner 1858 [22] and five lineages were recognized in *Pygocentrus nattereri* Kner 1858 in different river basins [23]. This underestimation of diversity is a consequence of the difficulty in identifying many species and their intra- and interspecific limits, due to the variation in body shape, sexual dimorphism, ontogeny [1, 4, 7, 24], and water color in Amazon basin [7, 25]. In addition, we highlight the scarcity of recent taxonomic revisions, with identification keys, of species-rich genera, such as *Metynnis*, *Myloplus*, and *Serrasalmus*.

From cytogenetic point of view, the greatest diversity is primarily related to diploid chromosome number (2n) and intra-and interspecific variations in the karyotype formula. In *Serrasalmus*, for example, three karyomorphs of *S*. *rhombeus* Linnaeus 1766 occurring in syntopy were found, which varied both in 2n and in the karyotype formula [26, 27]. Different karyomorphs were also observed in specimens of *S*. *maculatus* (described as *S*. *spilopleura* Kner 1858) from the Paraná-Paraguay basin [28] and the Amazon basin [29, 30].

Significant advances in cytogenetic studies in Serrasalmidae utilizing different approaches also occurred, ranging from chromosomal characterization of species, such as *Pygocentrus cariba* Humboldt and Valenciennes 1821 [31] and *Myleus micans* Lütken 1875 [32], to the use of cytogenetic markers to identify hybrids between *Colossoma* and *Piaractus* [33, 34]. Recently, the presence of a B chromosome restricted to females was described to *Metynnis lippincottianus* (Cope 1870) [35].

The 2n in the family varies from 54 chromosomes in *Colossoma* [36, 37], *Mylossoma* [38], and *Piaractus* [33, 38] to 62—in *Metynnis* [35, 39, 40]. Intermediate 2n, such as 58 and 60 chromosomes, were already reported in *Pygocentrus* and *Serrasalmus* [26, 28, 29, 37, 41–43]. However, little is known about this number within Myleini and other species of *Metynnis* (the first genus to diverge within Serrasalmini). Given the chromosomal diversity observed in Serrasalmidae, this study aimed: (i) to analyze cytogenetically species of Myleini and *Metynnis*, and (ii) using modern molecular phylogenies as a framework, propose a general pattern of chromosomal evolution within Serrasalmidae.

## Material and methods

In the present study, we analyzed a total of 39 specimens from eight species and three genera (Table 1). The sampling of specimens was authorized by Instituto Brasileiro do Meio Ambiente e Recursos Naturais Renováveis (IBAMA, License No. 28095–1) and the experimental procedure was approved by the Comitê de Ética na Utilização de Animais (CEUA) at Instituto Nacional de Pesquisas da Amazônia (INPA) (Approval No. 027/2017).

**Table 1:** Species and number of individuals analyzed, with their respective sampling locations and voucher number.

Species	Number of individuals Males Females	Location	Voucher
***Metynnis cuiaba* Pavanelli, Ota & Petry 2009**	0 1	Negro River (Anavilhanas Archipelago), AM 2°37’28.5”S, 60°58’16.8”W	INPA-ICT 59049
***Metynnis hypsauchen* (Müller & Troschel 1844)**	3 6	Uatumã River (Balbina Hydroelectric Dam), AM 1°55’02.2"S 59°28’23.7"W	INPA-ICT 59050
***Metynnis longipinnis* Zarske & Géry 2008**	1 2	Negro River (Anavilhanas Archipelago), AM 2°37’28.5”S, 60°58’16.8”W	INPA-ICT 59051
Myloplus asterias* (Müller & Troschel 1844)	1 1	Apeu floodplain, Castanhal (PA)	INPA-ICT 59052
***Myloplus lobatus* (Valenciennes 1850)**	3 0	Catalão Lake, AM 2°33’28.4”S, 60°46’29.7”W	INPA-ICT 59056
Myloplus rubripinnis* (Müller & Troschel 1844)	4 4	Guamá River, Belém (PA)	INPA-ICT 59053
Myloplus schomburgkii* (Jardine 1841)	9 3	Xingu River, Altamira (PA)	INPA-ICT 59054
Tometes camunani* Andrade, Giarrizzo & Jégu 2013	1 0	Belém (PA)	INPA-ICT 59055

AM = Amazonas state; PA = Pará state. / (*) Lacking precise geographic coordinates.

Cell suspensions were obtained from renal tissue, according to the protocol of [44]. C-banding was based on the [45] protocol, with minor modifications and stained with propidium iodide [46]. The extraction of total DNA was from muscle tissue, using the Wizard^®^ Genomic DNA Purification Kit (Promega), according to the manufacturer’s guidelines. The extracted DNA was quantified in 1.5% agarose gel with NanoVue^TM^ Plus (GE Healthcare). The sequences of 18S rDNA, 5S rDNA, and telomeric sequence were amplified by polymerase chain reaction (PCR), using the following primers: 18Sf (5’-CCG CTT TGG TGA CTC TTG AT-3’) and 18Sr (5’-CCG AGGACC TCA CTA AAC CA-3’) [47]; 5Sa (5’-TAC GCC CGA TCT CGT CCG ATC-3’) and 5Sb (5’- CAGGCT GGT ATG GCC GTA AGC-3’) [48]; and (TTAGGG)_5_ and (CCCTAA)_5_ [49]. Fluorescent in situ hybridization (FISH) was performed according to the protocol described by [50], but with modifications. The slides were denatured in 70% formamide/2xSSC at 70°C, and the spreads were dehydrated in an increasing ethanol series (70, 85 and 100%), for 5 min at each concentration. Subsequently, 20 μL of the hybridization mixture (100 ng of each probe, 50% deionized formamide, 20xSSC, and 10% dextran sulphate) was dropped onto each slide, and the mixture was hybridized at 37°C for 24 h in a moist chamber containing distilled water. The chromosomes were counterstained with DAPI (1.2 μg/mL) and mounted in antifade solution (Vector, Burlingame, CA, USA). The PCR products of the 18S rDNA gene and telomeric sequence were labelled by nick translation with digoxigenin 11-dUTP (Dig-Nick Translation mix; Roche) and 5S rDNA was labelled with biotin-14-dATP (Biotin Nick Translation mix; Roche), following the manufacturer’s instructions. The detection of the hybridization signals was performed with anti- digoxigenin -rhodamine (Roche Applied Science) for the 18S rDNA probes and the telomeric sequence, and with streptavidin (Sigma-Aldrich) for the 5S rDNA probes. Subsequently, the chromosomes were counterstained with DAPI, analyzed in an Olympus BX51 fluorescence microscope and classified according to [51]. The 5S and 18S rDNA sequences were obtained from the eight species present in Table 1, with addition to published data from [35].

For analysis of the evolution of the chromosome number in a phylogenetic context (adapted from [6]), data obtained from the eight species analyzed in this study were added to karyotype information already available in the literature *i*.*e*. [26, 29, 32, 33, 35, 37, 38, 42, 43].

## Results

**Fig 1:**
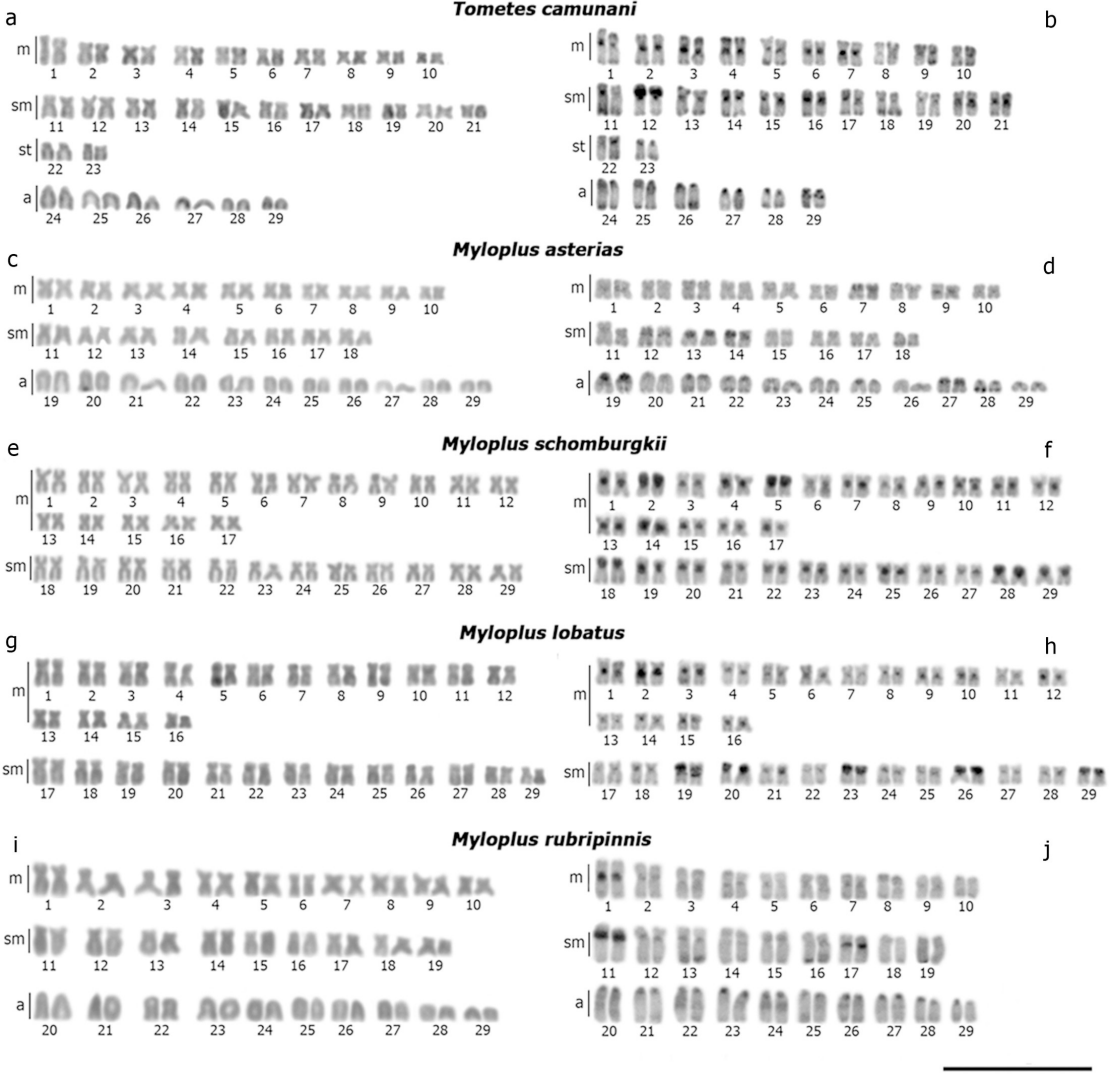
Karyotypes of the species with 2n = 58, stained with Giemsa (left column) and with C band (right column): (a, b) *Tometes camunani*; (c, d) *Myloplus asterias*; (e, f) *My*. *schomburgkii*; (g, h) *My*. *lobatus*; (i, j) *My*. *rubripinnis*. Scale bar = 10 μm.

**Fig 2:**
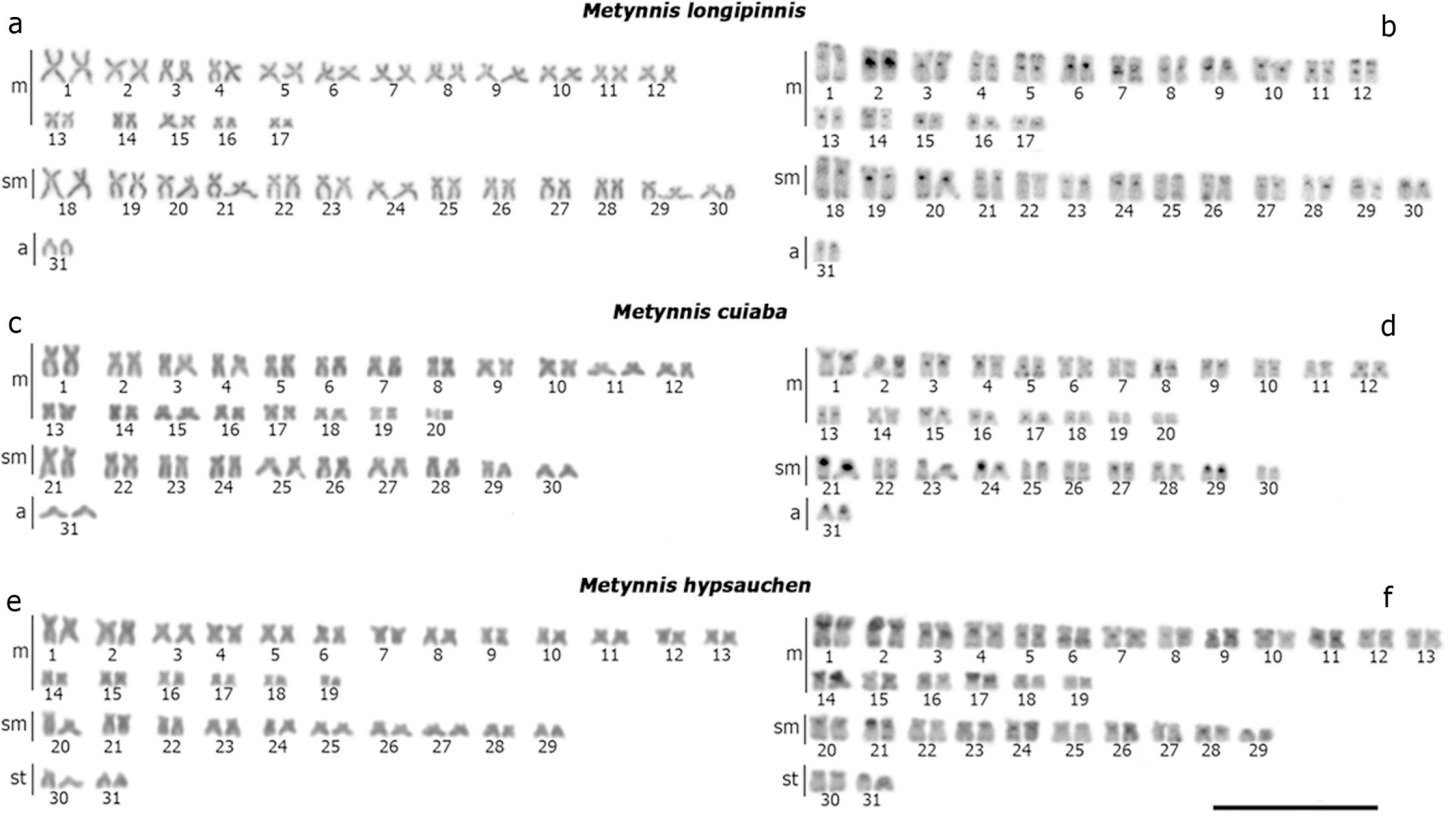
Karyotypes of *Metynnis longipinnis* (a, b); *Me*. *cuiaba* (c, d); *Me*. *hypsauchen* (e, f), stained with Giemsa (a, c, e) and C band (b, d, f). Scale bar = 10 μm.

The four species of the genus *Myloplus* and *T*. *camunani* have 2n = 58 chromosomes, while the species of *Metynnis* 2n = 62 chromosomes (Figs 1 and 2). The karyotype formulas and/or fundamental number (FN) are species-specific (Table 2).

**Table 2:** Species analyzed with their respective diploid and fundamental numbers, karyotype formula, and 18S and 5S rDNA position.

Species	2n	FN	KF	18S	5S
***Metynnis cuiaba***	62	122	34m+26sm+2a	8p; 29p	29p
***Metynnis longipinnis***	62	122	40m+20sm+2a	3p; 23q	3p; 31p
***Metynnis hypsauchen***	62	120	38m+20sm+4a	10p; 23q	10p; 23q
***Myloplus asterias***	58	94	20m+16sm+22a	13p; 19q; 25q	14q
***Myloplus schomburgkii***	58	116	34m+24sm	2p; 3p; 5p; 21p	19q
***Myloplus lobatus***	58	116	32m+26sm	2p; 8p; 22p	5q
***Myloplus rubripinnis***	58	96	20m+18sm+20a	20p; 22p; 24p	14q
***Tometes camunani***	58	104	20m+22sm+4st+12a	22p; 25p; 27p	2q

(2n = diploid number; FN = fundamental number; KF = karyotype formula); m = metacentric; sm = submetacentric; st = subtelocentric; a = acrocentric; p = short arm; q = long arm).

The distribution of constitutive heterochromatin occurred mainly in the pericentromeric regions of all chromosomes of all analyzed species, in addition to the presence of blocks in terminal portions of some chromosomes (Figs 1 and 2). Some species also had entire heterochromatic short arms in some pairs, such as pair 12 of *T*. *camunani* (Fig 1B), pairs 7, 13, 14, and the proximal region of pairs 19 and 27 of *My*. *asterias* (Fig 1D), pairs 2, 5, 18, and 28 of *My*. *schomburgkii* (Fig 1F), pairs 19, 20, 23, 26, and 29 of *My*. *lobatus* (Fig 1H), pair 11 of *My*. *rubripinnis* (Fig 1J), pairs 21 and 29 *Me*. *cuiaba* (Fig 2D), and the pairs 1, 2, 14, 17, 21 of *Me*. *hypsauchen* (Fig 2F). Also, heteromorphism of heterochromatic block size was observed in pair 2 of *My*. *lobatus* (Fig 1H) and pair 24 of *Me*. *cuiaba* (Fig 2D).

**Fig 3:**
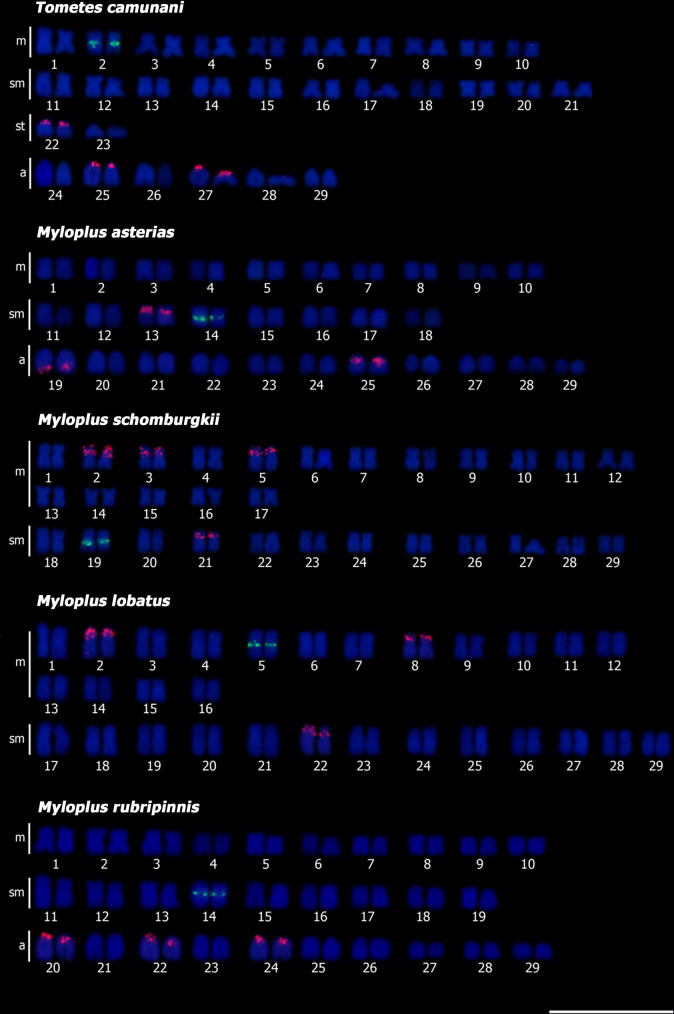
Karyotype of the five species analyzed by Double-FISH with 18S rDNA (red) and 5S rDNA (green) and counterstained with DAPI: (a) *Tometes camunani*; (b) *Myloplus asterias*; (c) *My*. *schomburgkii*; (d) *My*. *lobatus*; (e) *My*. *rubripinnis*. Scale bar = 10 μm.

The mapping of the 5S rDNA sequences showed only one pair with signal in the pericentromeric portion for the *Tometes* and *Myloplus* species, which were the pair 2 of *T*. *camunani* (Fig 3), 14 of *My*. *asterias* (Fig 3), and *My*. *rubripinnis* (Fig 3), 19 from *My*. *schomburgkii* (Fig 3) and 5 from *My*. *lobatus* (Fig 3). In relation to the 18S rDNA, the signals were found in three chromosomal pairs in four species: *T*. *camunani*, terminal portion, short arm, pairs 22, 25, and 27 (Fig 3), *My*. *asterias*, interstitial region of the short arm of pair 13, terminal region of the long arm of par 19, and pericentromeric portion of par 25 (Fig 3), *My*. *lobatus*, interstitial region of the short arm of pairs 2, 8, 22 (Fig 3), and *My*. *rubripinnis*, terminal region in pairs 20, 22, 24 (Fig 3). However, in *My*. *schomburgkii*, signals were seen in the interstitial region of the short arms of pairs 2, 3, 5, and 21 (Fig 3).

**Fig 4:**
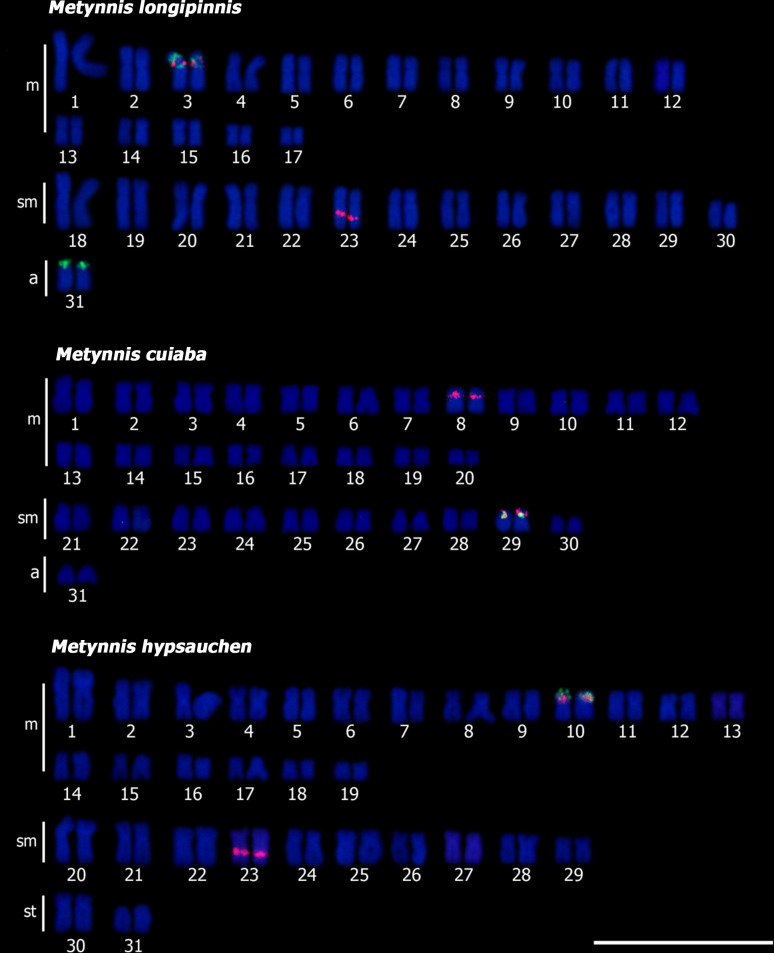
Double-FISH in *Metynnis*: (a) *Me*. *longipinnis*; (b) *Me*. *cuiaba*; (c) *Me*. *hypsauchen*, 18S rDNA (red); 5S rDNA (green) and counterstained with DAPI. Scale bar = 10 μm.

For *Metynnis*, the three species have synteny of the 18S and 5S rDNA in the interstitial portion of the long arms of the pairs 3 in *Me*. *longipinnis*, 29 in *Me*. *cuiaba*, and 10 in *Me*. *hypsauchen* (Fig 4). In *Me*. *longipinnis*, in addition to the aforementioned 5S marker, we detected another one in terminal portion of the short arm in pair 31 (Fig 4). Regarding the sites of the 18S rDNA, they were also observed in interstitial portion of the long arms of pair 23 in *Me*. *longipinnis* and *Me*. *hypsauchen* (Fig 4) and in the pericentromeric region in pair 8 in *Me*. *cuiaba* (Fig 4). Telomeric sequences were only detected in the terminal portions of the chromosomes in all species (S1 Fig).

**Fig 5:**
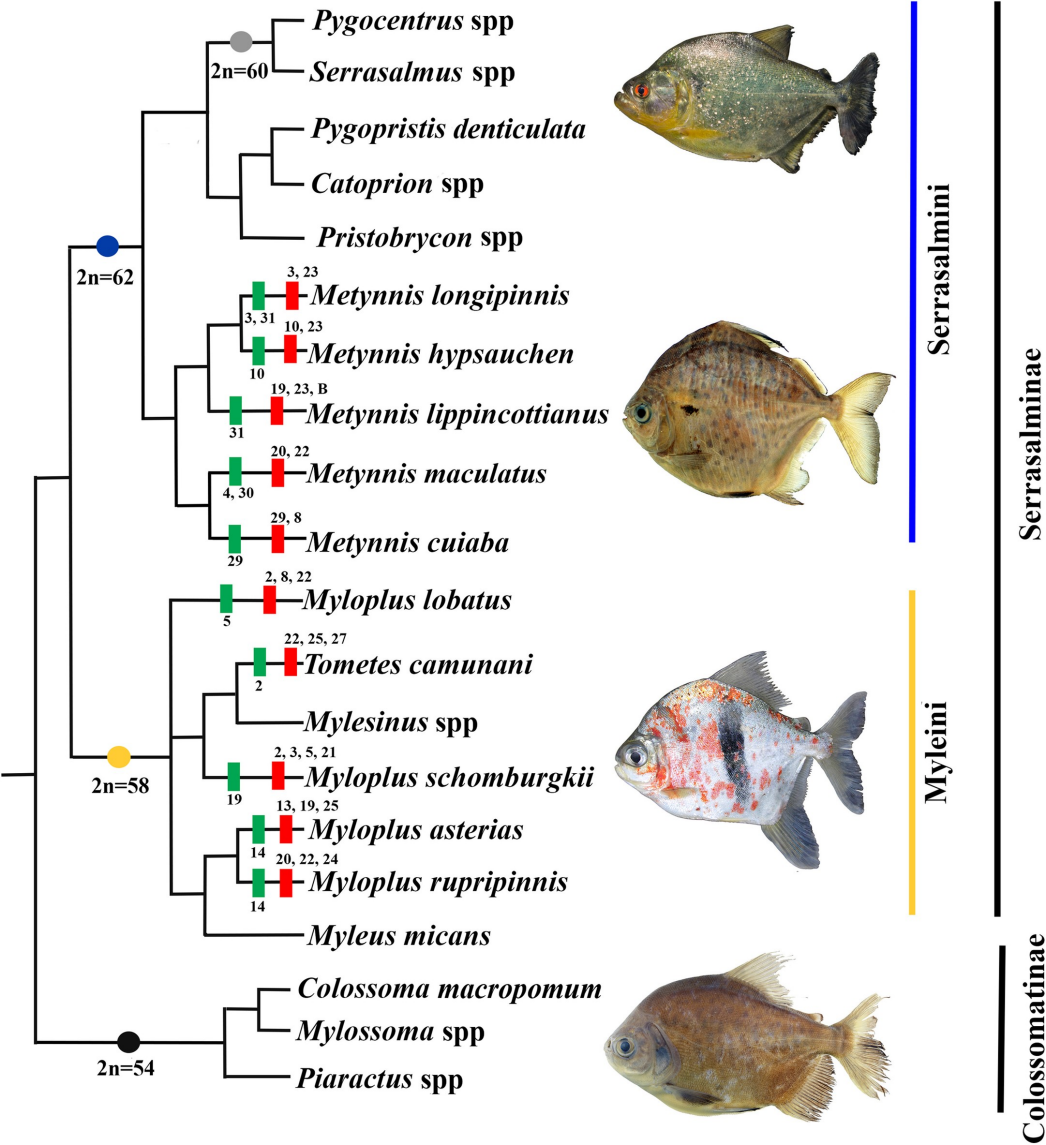
Cladogram adapted from Mateussi *et al*. [6], indicating the variation of the diploid number between the species and genera with available cytogenetic information, with the probable ancestral diploid number of the clades indicated by circles of different colors (2n = 54 black, 2n = 58 yellow, 2n = 60 gray, 2n = 62 blue). Data for *Pygocentrus* spp. and *Serrasalmus* spp. [26, 29, 37, 42, 43], and for *Metynnis* [35]. Data for Colossomatinae [33, 38]; and for *Myleus micans* [32]. Numbers indicate chromosomal pairs with 18S rDNA (red) and 5S rDNA (green).

The chromosome number within the main clades of Serrasalmidae was relatively stable. In Colossomatinae all species have 2n = 54, in Myleini: *Mylesinus*, *Myleus*, *Myloplus*, and *Tometes* possess 2n = 58 chromosomes, and in Serrasalmini: the species of *Serrasalmus* and *Pygocentrus* have 2n = 60 chromosomes (Fig 5).

## Discussion

The present study contributes to the knowledge about Serrasalmidae cytogenetics, mainly concerning Myleini, including for the first time species of the genera *Myloplus* and *Tometes*; and within Serrasalmini expanding to five the number of *Metynnis* species with chromosomal information. *Metynnis* was the first genus to diverge within Serrasalmini [6] and is essential to understand the evolution of remaining members of the tribe. The karyotype formulas and/or fundamental number were species-specific for the eight species, which can represent informative data to be used in combination with molecular markers to better understand the phylogenetic relationship in intergeneric level, especially concerning paraphyletic genera as *Myleus*, *Myloplus*, *Tometes*, and *Pristobrycon*.

We observed the presence of large heterochromatic blocks in the pericentromeric and terminal regions of some chromosomes in Myleini and in analyzed species of *Metynnis*. This pattern was already reported for other species of Myleini [33, 38] and Serrasalmini [26, 29, 35, 42]. The presence of fully heterochromatic short arms, as in *Myloplus schomburgkii* and *My*. *lobatus*, was also observed in *Serrasalmus compressus* Jégu, Leão & Santos 1991 and *S*. *elongatus* Kner 1858 [42] and is possibly related to interspecific variations in *Myloplus*. These heterochromatic blocks and arms may indicate chromosomal rearrangements such as the Robertsonian or non-R rearrangements, which can cause changes in 2n or not [52].

In the species analyzed in this study, some heterochromatic blocks are associated with ribosomal sequences mapped. In Serrasalmidae, this association was previously described in different species of *Serrasalmus* [37, 43], in *Colossoma macropomum* Cuvier 1816 and *Piaractus mesopotamicus* Holmberg 1887 [38], and in *Myleus micans* (Lütken 1875) [32]. In the case of piranhas, as *Serrasalmus rhombeus*, this association directly influenced the differentiation among karyomorphs [26], where the chromosomes, mainly subtelocentric/acrocentric, with 18S ribosomal cistrons, are C-band positive. These chromosomes are apparently those that underwent rearrangements, causing variations in the 2n and karyotype formulas, revealing that heterochromatin may be generating points that are susceptible to chromosomal breaks and contributing to the karyotype evolution of the group [26, 37].

The location pattern of the 18S and 5S rDNA sites registered in *Myloplus* and *Tometes* species resembles that already described in the family, with single 5S and multiple 18S labeling [37, 43]. To date, all analyzed *Serrasalmus* species also presented this 5S rDNA localization pattern. This pattern of ribosomal sequences can be used as a taxonomic marker, since both sites provide unique markers for each species analyzed. In addition, 5S rDNA was already reported as a relevant cytogenetic marker within the family, in which all species of *Serrasalmus* analyzed had this site in the interstitial region of pair 7 [37, 43]. Therefore, we recommend 5S rDNA to be used in integrative taxonomy approach of the family, along with DNA barcoding that is already being greatly employed in the past years [*e*.*g*. 6, 7, 53]. Despite the advances in species description of Serrasalmidae, 14 new taxa in the last decade [5], none of them included a *Serrasalmus* discovery, even with the several evidences of new species using COI [*e*.*g*. 21, 22].

On the other hand, the synteny between the 18S and 5S sequences, observed in the *Metynnis* species, had not yet been reported, and can be considered an unprecedented characteristic for the family. In addition to the syntenic pair, one additional chromosome pair presented 18S rDNA sites, coinciding with the number of markers for *Me*. *maculatus* (Kner 1858) and *Me*. *lippincottianus* [35], two very similar looking congeners, but not closely related according to molecular phylogenies [*e*.*g*. 6, 20]. The co-location of these cistrons in the three species seems to indicate that this condition is maintained and being propagated within *Metynnis*, suggesting some adaptive advantage for maintaining this organization in the genera, as suggested for some genera of Julidini (Perciformes) [54].

The data from the rDNA show an apparent conservation and organization within the clades/genera of the family. The 5S rDNA sequence is observed in pericentromeric portions in two pairs in *C*. *macropomum*, *Mylossoma* spp., and *Piaractus mesopotamicus* (Colossomatinae) [38], while in *Myleus micans*, *Myloplus*, and *Tometes* (Myleini), *Pygocentrus* and *Serrasalmus* (Serrasalmini) only one meta/submetacentric pair is a marker of this ribosomal site [32, 35, 37, 43]. On the other hand, for 18S rDNA an increase in the number of sites of the basal clade towards the derived clade can be observed. In Myleini, the species have 1 to 3 pairs of 18S bearers [33, 38, this study]. While in Serrasalmini all species of *Serrasalmus* and *Pygocentrus* have at least five pairs carrying these sequences, with a clear predominance in acrocentric chromosomes [37, 43].

In general, there is a similarity in the number of sites of the ribosomal sequences in each clade, which suggests that there is conservation of the chromosomal structure in Serrasalmidae. Although the mapping of telomeric sequences did not show any rearrangement, the variations in relation to the number and location of rDNA sequences between species, may indicate that these sequences have an evolutionary independence [55], between or within the genera of Serrasalmidae. As for example, in *Metynnis* with the presence of synteny in the three analyzed species, and with the homeology of the 5S rDNA in pair 7 of the *Serrasalmus* species [37, 43].

This conservation is also observed regarding the diploid chromosome number. The comparison to the phylogenetic relationship proposals of Mateussi et al. [6] and Kolman et al. [20], we observe that in Colossomatinae, all species have 2n = 54 [33, 38]. Within Serrasalminae, diploid chromosome number increased, the tribe Myleini (or Myleinae *sensu* Kolman et al. [20]) presented 2n = 58 (*Myleus*, *Myloplus*, and *Tometes*). In Serrasalmini (or Serrasalminae *sensu* Kolman et al. [20]), *Metynnis* species have 2n = 62 chromosomes, while, *Serrasalmus* and *Pygocentrus* have 2n = 60 chromosomes [26, 29, 37, 42, 43]. Previous hypotheses proposed that 2n = 54 chromosomes is the ancestral number of the family, with a tendency to increase the 2n number from 54 to 62 chromosomes [26, 29, 35, 38, 42]. The increase in diploid number would have occurred through chromosomal fission, since in Colossomatinae all chromosomes have two arms (meta and submetacentric type) while in the derived Serrasalmini there are some pairs of chromosomes with only one arm (acrocentric) [37, 38].

The increase in chromosome number in the tribe Myleini, along with a greater amount of chromosomes of subtelocentric/acrocentric types, is a condition that was already reported in different fish families. In Curimatidae, for example, most analyzed species have 2n conserved, equal to 54 meta/submetacentric chromosomes [52, 56–59]. It is interesting to note that the conservation of the diploid number (2n = 54) [17] can be considered a synapomorphy of Curimatoidea [60–64]. The only families of this clade, for which 2n diverged from 54 are Serrasalmidae [26, 32, 33, 37, 38, 67] and Curimatidae (*e*.*g*. *Potamorhina latior*, *P*. *altamazonica* and *P*. *squamoralevis*) [26, 33, 38, 56, 65–67], however they have 2n = 54 present in the species of the clades that first diverged.

In spite of the indication that the chromosomal fissions, coupled with the emergence of acrocentric chromosomes, are associated with an increase in the diploid number in Serrasalmidae, this change did not occur in a linear path from Myleini to Serrasalmini, since *Myloplus asterias*, *My*. *rubrippinis*, and *T*. *camunani* have 2n = 58, and several acrocentric chromosomes, while in *Metynnis*, the highest chromosomal number (2n = 62) is observed, and a larger number of the meta- and submetacentric, and fewer acrocentric chromosomes. This suggests that, in addition to fission, other rearrangements, such as fusions, translocations and pericentric inversions, were involved in the evolution of these species and modified the 2n and karyotype formulas among the clades. These rearrangements occurred in a dynamic and complex way, independently in the different clades, since each of them has unique characteristics, as the synteny present in *Metynnis* and the homeology of the 5S rDNA pair in *Serrasalmus*.

Therefore, the chromosomal macrostructure of the Serrasalmidae species is conserved within the main clades, with higher variation in Serrasalmini. This fact makes the family a very interesting group to study, because the different karyotype formulas and locations of ribosomal sequences, recorded in some species can be used as cytotaxonomic markers and assist in the identification of species, given the difficulty and taxonomic uncertainties that still persist in Serrasalmidae, despite all these advances. Furthermore, the diversity of chromosomal markers highlights the importance of integrating cytogenetic studies with systematic studies, whether they are morphological or molecular. The expansion of both chromosome studies and the number of localities sampled would contribute further to confirm the evolutionary process that occurred in Serrasalmidae and also to corroborate the diversity of species in the different clades.

## Supporting information

S1 Fig Metaphases of eight species of Serrasalmidae analyzed by FISH using a telomeric probe and counterstained with DAPI. (a) *T*. *camunani*; (b) *M*. *asterias*; (c) *M*. *schomburgkii*; (d) *M*. *lobatus*; (e) *M*. *rubripinnis*; (f) *M*. *longipinnis*; (g) *M*. *altidorsalis*; (h) *M*. *hypsauchen*. Scale bar = 10 μm.
(TIF)
