# Supplementary figures and images for: Comparative cytogenetics of Serrasalmidae (Teleostei: Characiformes): The relationship between chromosomal evolution and molecular phylogenies
Source: PLoS One. 2021 Oct 7;16(10):e0258003. doi: 10.1371/journal.pone.0258003 (PMC8496811; doi:10.1371/journal.pone.0258003)

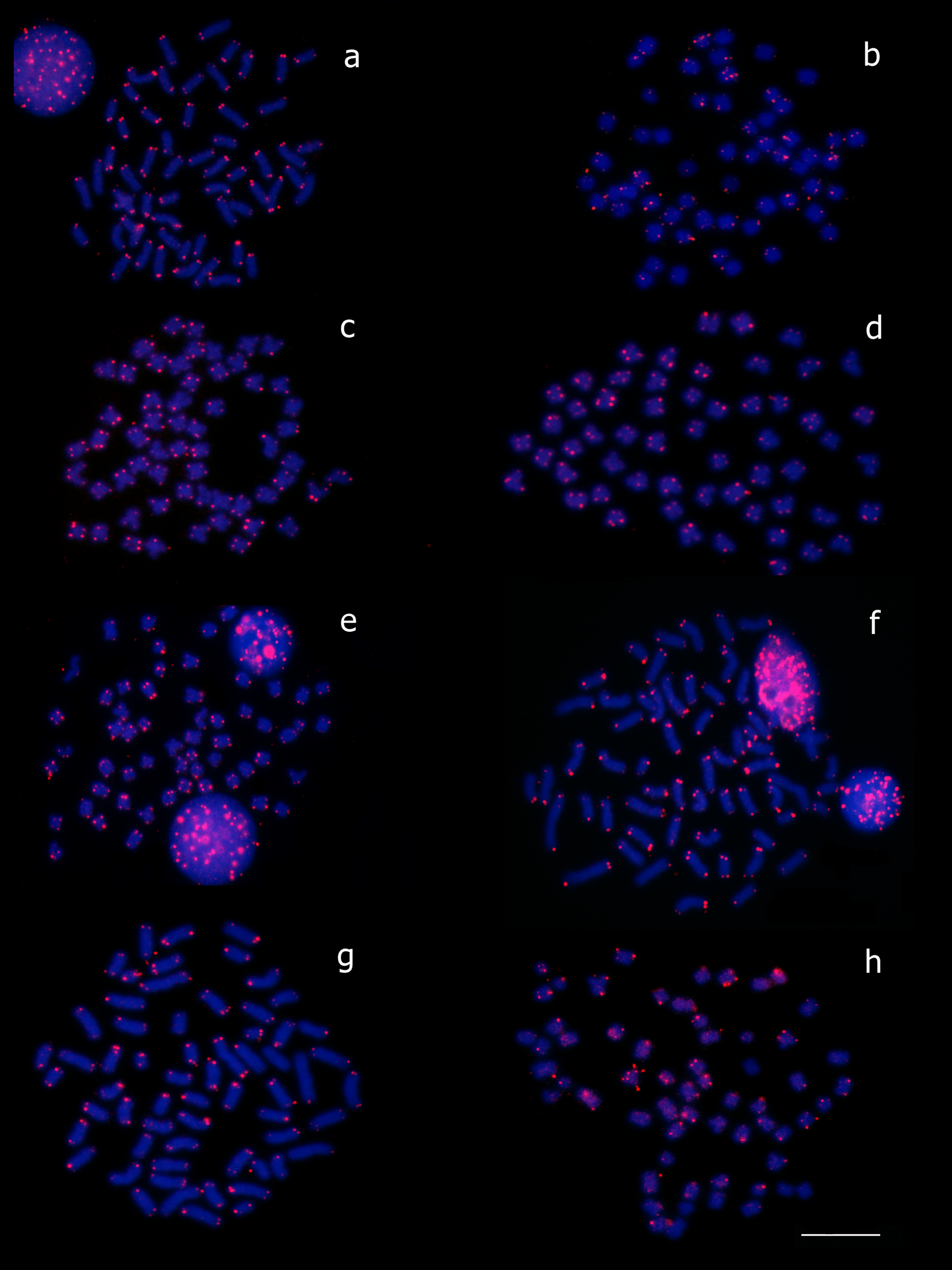

Supplement: S1 Fig — (a) T. camunani; (b) M. asterias; (c) M. schomburgkii; (d) M. lobatus; (e) M. rubripinnis; (f) M. longipinnis; (g) M. altidorsalis; (h) M. hypsauchen. Scale bar = 10 μm. (TIF) [file pone.0258003.s001.tif]
